# What is ‘Spiritual Health’? A Survey of Social Prescribers in the United Kingdom

**DOI:** 10.1007/s10943-025-02488-z

**Published:** 2025-11-14

**Authors:** Ishbel Orla Whitehead, Amy O’Donnell, Barbara Hanratty

**Affiliations:** Population Health Sciences Institute, Faculty of Medical Sciences, Newcastle upon Tyne, UK

**Keywords:** Spiritual health, Social prescribing, Spirituality, Primary care, Consultation tools

## Abstract

Social prescribing aims to provide holistic care to patients beyond that offered in consultations with clinical health professionals. Support may be directed at many aspects of health and well-being, including spiritual health. Spiritual health is known to be associated with many other physical, mental and rehabilitation health outcomes. How social prescribers define and understand ‘spiritual health’ is unknown. In this survey, we aim to explore what UK social prescribers understand by the term “spiritual health.” We asked social prescribers: ‘what does spiritual health mean to you?’ Responses were analysed using descriptive statistics. Free text data were subject to deductive thematic analysis using a priori themes from the literature on definitions of spiritual health. One hundred and seventy-one social prescribing link workers participated; 153 gave a definition of spiritual health. Twice as many participants described themselves as spiritual than described themselves as religious. Definitions of spiritual health fell into three themes: 1. A sense of self, peace, meaning and purpose; 2. Connections to others, the world, or a deity; 3. Spiritual or religious practice. Spiritual health appears to be a term with meaning to UK social prescribers, distinct from religiosity. Use of this ‘working definition’ of spiritual health will allow further research into how spiritual health fits within social prescribing in the UK to move forwards.

**Graphical Abstract:**

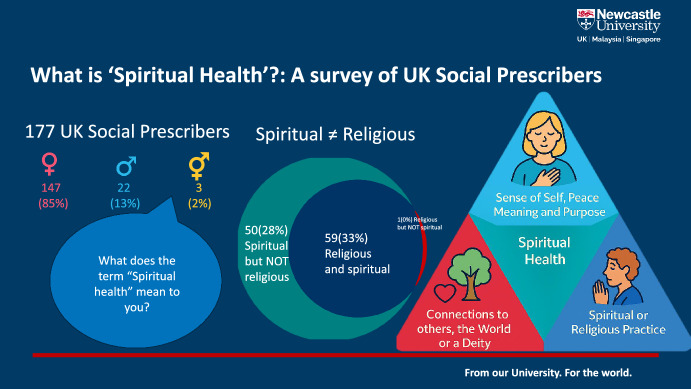

**Supplementary Information:**

The online version contains supplementary material available at 10.1007/s10943-025-02488-z.

## Introduction

Social prescribing is a primary care-based approach that addresses non-medical determinants of health by connecting individuals with community-based, non-clinical resources and activities (National Academy for Social Prescribing (2022); Calderón-Larrañaga et al., 2024). Its purpose is to enhance health and well-being through a holistic focus on salutogenesis, or health creation (Calderón-Larrañaga et al., 2024; Wildman & Wildman, 2021; Wood et al., 2021). Social prescribing encompasses spiritual aspects of health—for example, by fostering meaning, purpose, and connectedness (Calderón-Larrañaga et al., 2022), and through “green” or “blue” prescribing that promotes engagement with nature (Alejandre et al., 2023; Leavell et al., 2019). However, to date there has been little exploration of how social prescribing explicitly intersects with the concept of “spiritual health.”

Spiritual health consistently emerges as a significant determinant of health. Epidemiological studies demonstrate that religiosity and spirituality are associated with longevity, even after adjusting for demographic and lifestyle factors (Dominguez et al., 2024; Powell et al., 2003; Snider and Mcphedran (2014); Zimmer et al., 2016). Benefits have been observed across a range of conditions relevant to primary care, including mental health (Gonçalves et al., 2015), HIV (Litalien et al., 2022), dementia (Agli et al., 2015), cardiovascular and respiratory disease (Abu et al., 2018; Luskin, 2000), cancer (Jim et al., 2015), and occupational burnout (Whitehead et al., 2022a, 2022b). Conversely, unmet spiritual needs can be detrimental, contributing to greater pain and higher mortality (Grant et al., 2004; Harris et al., 2018; Koenig, 2012; Pargament et al., 2001; Rodin et al., 2009). The COVID-19 pandemic, with its associated distress, has arguably heightened the relevance of spiritual health (Heidari et al., 2020; Lucchetti et al., 2020; Safarabadi et al., 2024; Upenieks, 2022).

Despite its importance to holistic, whole-person care (Yang et al., 2016; Zimmer et al., 2016), “spiritual health” remains ambiguously defined, with no universally accepted definition (Appleby et al., 2018; Jaberi et al., 2019; Saunders et al., 2020; Whitehead et al., 2021). Spirituality is understood in diverse ways (Koenig, 2008; Saad et al., 2022), and meanings may evolve across the life course (Tanyi et al., 2009). Spirituality and religiosity (e.g., participation in religious practice) are often conflated (Daaleman & Nease, 1994; Koenig, 2008; Larimore, 2001; McCauley et al., 2005; Monroe et al., 2003; Saguil & Phelps, 2012), yet many authors treat spiritual health as distinct from religiosity (Saunders et al., 2020; Whitehead et al., 2021; Zimmer et al., 2016). A UK survey of primary care doctors identified three broad themes in how spiritual health is understood: self-actualisation and meaning, transcendence and relationships beyond the self, and expressions of spirituality (Whitehead et al., 2021). While most recognised its relevance, many did not consider it part of their clinical role. Nonetheless, spiritual health care—integral to holistic care—may be supported through social prescribing (South Kent Mind; Whitehead et al., 2022a, 2022b).

Social prescribing is a growing policy priority in UK primary care, designed to personalise care and reduce pressure on the NHS (Husk & Sanderson, 2024; NHS England, 2025). At its centre are link workers, who act as referral points for general practitioners and other professionals to connect patients with non-medical, socially based interventions (Husk & Sanderson, 2024). Link workers, embedded in their communities, facilitate access to services, such as crafts, choirs, grief support, and community mental health groups. Their numbers are expanding through government investment (Husk & Sanderson, 2024; NHS England, 2025). The aim is to provide personalised, relational care that addresses patients’ holistic needs (Griffith et al., 2022).

The Social Prescribing model was originally developed around the concept of salutogenesis—the “creation of health” (Health Education England, 2016; Lindström & Eriksson, 2006)—which includes the spiritual dimension. However, current UK initiatives make little explicit reference to spiritual care providers or faith communities (Brandling & House, 2009; Health Education England, 2016; Polley et al., 2017). While clinicians may not feel best placed to address patients’ spiritual needs directly, social prescribing may offer a pathway (Whitehead et al., 2022a, 2022b). Importantly, little is known about how link workers themselves understand spiritual health, or how they incorporate it into daily practice. Given the expanding role of social prescribing in UK primary care, this represents a key gap in knowledge.

## Method

An online survey was distributed to social prescribing workers (this included a range of job titles), using JISC online surveys. (Jisc Online Surveys, 2023) The survey was sent to health service (NHS) networks to be included in newsletters, as well as professional online groups, and forwarded to practice managers and GP practices directly. Written consent was taken online, prior to the start of the online survey. Research ethics committee approval was obtained from Newcastle University (April 2024, 45,810/2023) and the Health Research Authority (IRAS number 343749). This study is grounded in a pragmatic research paradigm. This study is part of a larger study (the SHARP study, Spiritual Health Awareness and Recommendations in Primary care).

Questions collected demographic data from the participants, including gender, ethnicity and belief, and occupational characteristics. (Appendix 2).

Participants were asked to rate the following statements using a five-option Likert scale:

- I am a spiritual person
- I am a religious person
- I am spiritually healthy

They were also asked to contribute free text in responses to the question ‘what does the term ‘spiritual health’ mean to you?’.

### Data Analysis

#### Quantitative

Data were collated by the JISC survey software (Jisc Online Surveys, 2023) and analysis was conducted using the Stata MP 18.0 package. (StataCorp., 2023) Associations were assessed using McNemar’s test, if appropriate. Data were aggregated where small numbers require it for statistical analysis, and to preserve participant anonymity.

#### Qualitative

Free-text responses to the question ‘what does the term ‘spiritual health’ mean to you’, were analysed using a deductive thematic analysis, based on a priori themes developed by immersion in the literature (Box 1). A four-step process was used:(13) immersion in the data; stratifying to identify themes by comparing and contrasting similar codes; review of categories; and finally drawing these together to identify the central themes. One researcher analysed the data and discussed the findings with the research team. Outlying cases were examined, to identify insights from those most and least comfortable with the topic. The researchers were cognisant that their approach to the topic would be influenced by their own identities, cultures and religious backgrounds in their approach to the topic.

**Table:** Box 1. Definitions of spiritual health used in research

Humanity- spirituality, the spirit or the soul is part of the universal human experience (Puchalski, 2014; Saguil & Phelps, 2012; Whitehead et al., 2021)
Meaning and purpose, or self-actualisation (Larimore et al., 2002; Mueller et al., 2001; Paal et al., 2015; Puchalski, 2013, 2014; Rumbold, 2007; Whitehead et al., 2021)
Inner strength, peace, and resilience (Paal et al., 2015; Whitehead et al., 2021)
Transcendence, or a sense of connectivity to aspects outside the self, for example with the community, society, family and nature (Larimore et al., 2002; Mueller et al., 2001; Puchalski, 2014; Rumbold, 2007; Whitehead et al., 2021)
Relationship with the divine or sacred (Puchalski, 2014; Whitehead et al., 2021)
Expression- beliefs, values, traditions, rituals and practices (Mueller et al., 2001; Saguil & Phelps, 2012; Whitehead et al., 2021)

### Public And Patient Involvement (PPI)

From the early stages of development of this project, members of VOICE, a network of public, patients and carers (https://www.voice-global.org/about/) were involved in shaping the direction of the research. Participants had expressed mixed views about the topic, and there appeared to be a degree of stigma regarding spiritual health needs. Participants felt that spiritual health may not be a doctor’s role, however all participants asserted that holistic, humanitarian care was essential.

## Results

**Fig. 1:**
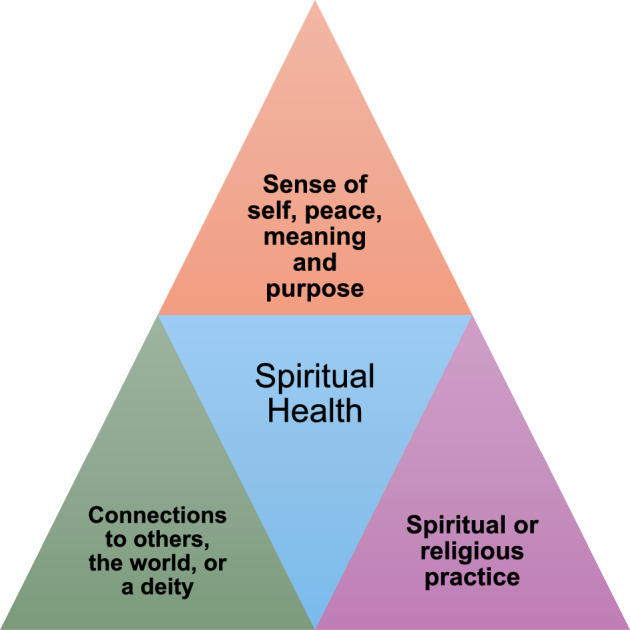
Definitions of spiritual health offered by social prescribers

One hundred and seventy-one social prescribing workers responded (Table 1). The majority were white (88%) and female (85%). While recruitment from England was good, few participants were recruited from the other UK nations, despite targeted efforts such as telephone calls and additional email invitations. The majority (112) of respondents used terms, such as social prescribers, link workers, or similar to describe themselves. However, respondents also called themselves care coordinators (19), coaches (6) and other titles such as community connector or navigator. Twice as many participants described themselves as spiritual than religious (127 agreed they were spiritual, 63 agreed they were religious) *χ*^2^ = 30.4, *p* < 0.05 (Table 2). 50 (28%) of participants described themselves as spiritual, but did not describe themselves as religious, suggesting these are different things. One hundred and fifty-four participants gave a free text definition of the term ‘spiritual health’, varying in length from a few words to a list of bullet points and paragraphs. Definitions fell into three themes: sense of self, peace, meaning and purpose; connections to others, the world, or a deity; spiritual or religious practice (Fig. 1).

**Table 1:** Participant demographics

Characteristic (*n* = 171)	Number of participants	Percentage
Total	171	
Gender		
Female	146	85
Male	22	13
Not disclosed or self-described	3	2
Ethnicity		
White background	151	88
Asian background	10	6
Black background	4	2
Mixed or multiple backgrounds	5	3
Belief related group		
Christian	76	44
No religion	70	41
Muslim	9	5
Humanist	4	2
Spiritual, not religious	4	2
Other	2	1
Prefer not to say	5	3
Area of UK		
South West of England	39	23
East Midlands	25	15
North East England	21	12
South East England	21	12
Yorkshire and the Humber	18	11
East of England	18	11
London	15	9
North West England	8	5
Other areas of England	2	1
Scotland, Northern Ireland or Wales	4	2

**Table 2:** Are participants religious or spiritual?

	Number of respondents (percentage)				
	Strongly agree	Agree	No opinion	Disagree	Strongly disagree
I am a spiritual person	57 (33%)	70 (41%)	18 (11%)	19 (11%)	7 (4%)
I am a religious person	22 (12%)	41 (24%)	27 (16%)	48 (28%)	32(19%)
I am spiritually healthy	33 (20%)	82 (49%)	37 (22%)	11 (7%)	5 (3%)

### Sense of Self, Peace, Meaning and Purpose

Throughout the survey, the social prescriber participants noted repeatedly their individual, person-centred approach to their work. This was shown clearly in their definitions of spiritual health. Importance of the individual, fidelity to a sense of identity, and personal ethical and moral codes, were key to the majority of definitions, especially for those participants who expanded at length. A concept of being a good person with a strong sense of meaning and purpose appears to be a key part of spiritual health. Participants mentioned a sense of coherence, balance and peace as core to good spiritual health. Good spiritual health appeared to represent the integration of multiple factors of self and identity into a strong sense of self-awareness and morality, the specific aspects of that sense appears to vary between individuals. This sense of self also fed into the second theme of connections beyond the self.

Spiritual health means being comfortable with yourself, being curious about the world around you and wanting to make continued improvements to the person you are, and how you interact with others. It means being kind to yourself and understanding you can't always be happy but that there is value in experiencing all kinds of emotions. Participant 122

For me, spiritual health is about how I consider myself and how I put myself forward in the world. It is important to me that I am a good person who does things to help and support the people I come into contact with. Participant 111

A few participants explained that aligning with a particular moral code or religion was important to their identity, and sense of self; however, most participants described spiritual health as something that existed outside one specific religion.

As a Christian, and if speaking to someone with faith, I would say spiritual health is an understanding of God, the world and our relationship with both as well as with ourselves (Our identity in Christ - if Christian) Participant 134

### Connections to Others, the World, or a Deity

Following from having a strong sense of self, participants then went on to emphasise connectivity to others as another core aspect of spiritual health. Some mentioned connections and relationships with a deity, or higher power. Many described their relationships more generally within communities or a sense of being part of the wider world, or connections to ‘something bigger’. One participant gave a lengthy definition that covered most aspects of spiritual health; they included transcendence and connection as differing concepts to them.

Being kind and generous, consideration of others, offering a service to support others. Connection with nature, animals, environment. Rejecting discrimination, disrespect of others. Holding boundaries. Participant 32

Participants described positive relationships as being part of ‘spiritual health’:

The term 'spiritual health' is very much linked into one's relationship to Jesus (i.e. not religion), and how the current state of that relationship is at any given point in time. Participant 123

Spiritual health for many would mean connection with self and the sense of 'beyond' - be that another deity/deities, a greater power, the other or a sense of the deeper connection with self or the earth. Participant 134

In contrast, however, one participant focused on their need for solitude:

being able to relax, unwind and be me. …holidays alone without the family. Participant 41

### Spiritual or Religious Practice

Participants shared their own practices they felt that were beneficial to their spiritual health, such as nature walks, devotions, deep breathing exercises, oracle cards, or meditation, as well as those they felt could be relevant to others—attendance at places of worship, yoga, prayer. Participants appeared to value highly, any practices that they were aligned with, and there appeared to be a bias towards practices such as meditation and yoga.

To me, spiritual health means being at peace with oneself, and be able to self sooth, through whatever medium suits the individual. My spiritual practice comes in the form of nature therapy and meditation. Participant 171

I find spiritual practices such as yoga and meditation easier to explore/suggest to patients, perhaps because it aligns with my beliefs. Participant 82

Mindfulness, yoga, attending church or having beliefs. Taking time for you and your wellbeing in an holistic way. Participant 157

### Synonymous with Religiosity

The majority of participants had a definition that did not mention religion, and some distinguished spiritual health from religiosity, or described it as sometimes including religion, but sometimes not. While many participants referred to ‘breathing’ activities, yoga or meditation, these appear to be divorced from their religious roots. A small number of participants described spiritual health only in religious terms, with one participant quoting six verses from the Bible to expand their definition of spiritual health from that lens.

Having a spiritual belief (religious or non-religious) that helps you focus, feel guided, and have a sense of belonging and belief. Participant 36

In my opinion it means to do with religion and faith and what you believe in. Participant 23

For me it means are you a Christian and have you been born again. Participant 6

Non-religious. Sense of well-being. Appeals to someone who may be interested in alternative therapies. Participant 154

Much more comfortable discussing yoga meditation etc, religion feels more intrusive Participant 132

### No Meaning

A small number did not give a definition because they felt the term was without meaning for them. The numbers were too small to analyse any further.

In all honesty it's not something that I think about Participant 138

Nothing to me personally as I have no religious beliefs. Participant 151

## Discussion

### Summary of Main Findings

This is a novel study that uses a survey of UK social prescribers to explore their understanding of the term “spiritual health.” We found that some social prescriber respondents equated spiritual health with religiosity, especially if they themselves were religious. However, it is notable that twice as many participants described themselves as spiritual compared to those who were religious, suggesting that many participants see spiritual and religious as separate concepts. The vast majority of participants offered a sentence, and a few offered small essays, as to what spiritual health meant to them. This indicates that, despite it being perceived as difficult to define, the term was meaningful to most participating social prescribers. It is unclear whether the few participants who did not offer a definition, felt that it was meaningless. Spiritual health as a term appears to have meaning to UK social prescribers, encompassing a sense of self, peace, meaning and purpose; connections to others, the world, or a deity; spiritual or religious practice.

There appeared to be a bias towards more ‘modern’ (in the West) aspects of spiritual health as being more acceptable. For example meditation, yoga, mindfulness were often viewed as universally beneficial, whereas prayer, or religious practice, was viewed as more taboo and personal, despite practices such as mindfulness having religious roots (Brown, 2016). This has been found in other UK reports (Rozario & Platt, 2025). This bias could impact on the recommendations social prescribers make when discussing spiritual health with patients, and should be researched further.

### Comparison with other Literature

Although evidence shows that social prescribers value salutogenesis, (Rakel, 2008) the creation of overall health, which includes the spiritual, spiritual health is often felt to be undefined. This is the first time that social prescribers have been asked explicitly to define spiritual health in research. The themes identified mirror those found previously with UK GPs (Whitehead et al., 2021). While other authors have argued that spiritual health is synonymous with religiosity (Koenig, 2012), it appears within the UK context that social prescribers and GPs think of these as different concepts (Whitehead et al., 2021), While there is ongoing work looking at how faith and social prescribing interact (Rozario & Platt, 2025), our project is the first to look at wider spiritual health beyond faith definitions in social prescribing. In their definitions participants covered all the areas of spiritual health established in the literature (Box 1). However, they placed great emphasis on peace, meaning, purpose, and the use of breathing as a spiritual practice.

While there has been research into definitions of spiritual health in other groups, e.g. palliative care, nurses, general practitioners/family doctors (Box 1), (Jaberi et al., 2019) we have not found any previous published work with social prescribers. However, there does appear to be a cross-disciplinary consensus that the term has a meaning that encompasses common themes of the self, meaning, purpose, ethics and peace; relationships and transcendence; practices.

### Strengths and Limitations

Recruiting social prescribers via newsletters and other forms of publicity produced a reasonable response, however respondents represent a subsection of the overall body of social prescribers in the UK (National Academy for Social Prescribing, 2023). We have no precise information on the denominator population or those who decided not to take part. The range of views that were expressed provides some confidence in our approach, but we acknowledge that people with strong views from either the ‘rejecting’ or ‘embracing’ ends of the spectrum may be more likely to have participated. Hence, we do not know if the high levels of identification as a ‘spiritual person’ was particular to the respondents, or reflective of the wider social prescribing population.

Participants were mostly female and white, and while this may reflect the profile of a majority of British social prescribers there are no national data to confirm this. The majority of respondents were from England, despite targeted efforts. We do not anticipate that there would be large national variation in social prescriber characteristics within the UK, and the response rate may reflect variation in social prescribing capacity in the different nations. Non-Christian religions were combined due to small numbers, limiting analysis of the effect of religious affiliation.

### Implications and Further Research

Spiritual health appears to be a meaningful term to social prescribers, and many articulated meanings both in their personal lives and practice, as well as meaning to others and those they worked with. While the term ‘spiritual health’ remains personalised, and may mean different things to different people, this study gives a useful ‘working definition’ to allow further research into how spiritual aspects of health fit into the salutogenesis involved in social prescribing.

## Conclusion

Our work suggests that a majority of social prescribers find meaning in the term ‘spiritual health’. They are able to offer a definition that is congruent with those found in the international literature and previously described in primary care. Understanding of spiritual health was distinguished from religion and religious practice by most, to include meaning, purpose and connections to others. The findings provide a useful “working definition” to allow further research into how spiritual health should best fit within social prescribing.

## Supplementary Information

Below is the link to the electronic supplementary material.

Supplementary file1 (PDF 768 KB)

## Data Availability

Data are saved on Newcastle University secure servers and may be available in negotiation with the first author. While participants were not consented to allow public sharing of this data, data are available upon reasonable request to the authors.
